# Nasopharyngeal Microbiota Profiling of SARS-CoV-2 Infected Patients

**DOI:** 10.1186/s12575-020-00131-7

**Published:** 2020-07-25

**Authors:** Flavio De Maio, Brunella Posteraro, Francesca Romana Ponziani, Paola Cattani, Antonio Gasbarrini, Maurizio Sanguinetti

**Affiliations:** 1 Dipartimento di Scienze di Laboratorio e Infettivologiche, Fondazione Policlinico Universitario A. Gemelli IRCCS, Largo A. Gemelli 8, 00168 Rome, Italy; 2 Dipartimento di Scienze Gastroenterologiche, Endocrino-Metaboliche e Nefro-Urologiche, Fondazione Policlinico Universitario A. Gemelli IRCCS, Rome, Italy

**Keywords:** Nasopharynx, Bacterial community, SARS-CoV-2 infection, 16S rRNA sequencing

## Abstract

We analyzed the bacterial communities of the nasopharynx in 40 SARS-CoV-2 infected and uninfected patients. All infected patients had a mild COVID-19 disease. We did not find statistically significant differences in either bacterial richness and diversity or composition. These findings suggest a nasopharyngeal microbiota at least early resilient to SARS-CoV-2 infection.

## Introduction

The human upper respiratory tract is the major portal of entry for infectious droplet or aerosol-transmitted microorganisms, including the 2019 emerged severe acute respiratory syndrome coronavirus 2 (SARS-CoV-2) [1]. Consistent with an ongoing rise in the number of coronavirus disease 2019 (COVID-19) cases worldwide, the updated World Health Organization estimates on 29 April 2020 reported 3,023,788 confirmed cases, including 208,112 deaths, over time (https://covid19.who.int/). Because of apparently absent cross-protective immunity from related viral infections, SARS-CoV-2 transmissibility is high, hence facilitating widespread person-to-person transmission [2]. Both asymptomatic and symptomatic patients may transmit the virus [3], consistent with similar viral loads detected in the nasal (sampled from the nasopharynx) and throat swabs from both patients [4].

As with influenza virus, respiratory samples provide the greatest yield of SARS-CoV-2 nucleic acid shedding [4, 5]. In one study analyzing nine patients with mild courses of COVID-19, swab samples taken during the first week of symptoms displayed high loads and successful isolation of the virus and, importantly, the presence of viral replicative RNA intermediates confirmed the active replication of SARS-CoV-2 in the throat [5]. The virus uses the host receptor angiotensin-converting enzyme 2 (ACE2), predominantly expressed in the lung, to interact with its spike protein [6]. This interaction would be responsible of the extension of tropism to multiple tissues [7], which include the throat tissue with anyhow low ACE2 expression [5], following a fusion activity gain of SARS-CoV-2 spike protein at the S1-S2 junction that is not present in SARS-CoV [8].

Unlike influenza virus [9–12], no studies have explored the relationship between SARS-CoV-2 infection and the bacterial community (also referred to as microbiota) within the nasopharynx. Therefore, we investigated whether the presence of the virus in the nasopharynx might reflect alterations of the resident microbiota by comparing the bacterial communities of SARS-CoV-2 infected and uninfected patients.

## Methods

We collected nasopharynx samples from patients who were (*n* = 18) or were not (*n* = 22) diagnosed with COVID-19 based on the SARS-CoV-2 RNA detection using a real-time reverse transcriptase-polymerase chain reaction (RT-PCR) assay [13]. This study received appropriate ethical review committee approval, with a waiver of informed consent.

Patients with samples collected based on the clinical COVID-19 suspicion (i.e., who presented symptoms of acute respiratory infection in the emergency department of the Fondazione Policlinico Universitario A. Gemelli (FPG) IRCCS Hospital of Rome, Rome, Italy) [14] from March 6 through March 9, 2020, were included. Nasopharyngeal swabs were tested for RT-PCR based COVID-19 diagnosis [13] using the Korean Ministry of Food and Drug Safety approved Allplex 2019-nCoV assay (Arrow Diagnostics S.r.l., Genova, Italy). The same samples were used to profile the nasopharyngeal microbiota in all 40 patients concomitantly.

Genomic DNA was extracted using the QIAGEN EZ1 Advanced XL system and the V5–V6 hypervariable region of the bacterial 16S rRNA gene was sequenced using an Illumina MiSeq V2 chemistry (2 × 250 bp) as described [11]. Sequencing data were processed for alignment and quality filtering in QIIME2 v2019.1 [15, 16], and representative amplicon sequence variants were obtained by the DADA2 algorithm [17] available at 10.1038/s41587-019-0209-9. Samples were rarefied to the minimum number of sequence reads obtained totally (range, 107,933–165,427) to perform subsequent analyses. Taxonomic annotation was performed using both VSEARCH [18] and SILVA database v132 [19]. Statistical tests, including Wilcoxon signed rank test and permutational analysis of variance (PERMANOVA) were performed in R software (R Foundation for Statistical Computing, version 3.6.0).

No patients developed pneumonia as documented in the absence of significant abnormalities (i.e., multifocal ground-glass opacities) on chest computed tomography (CT) images.

## Results

**Fig. 1:**
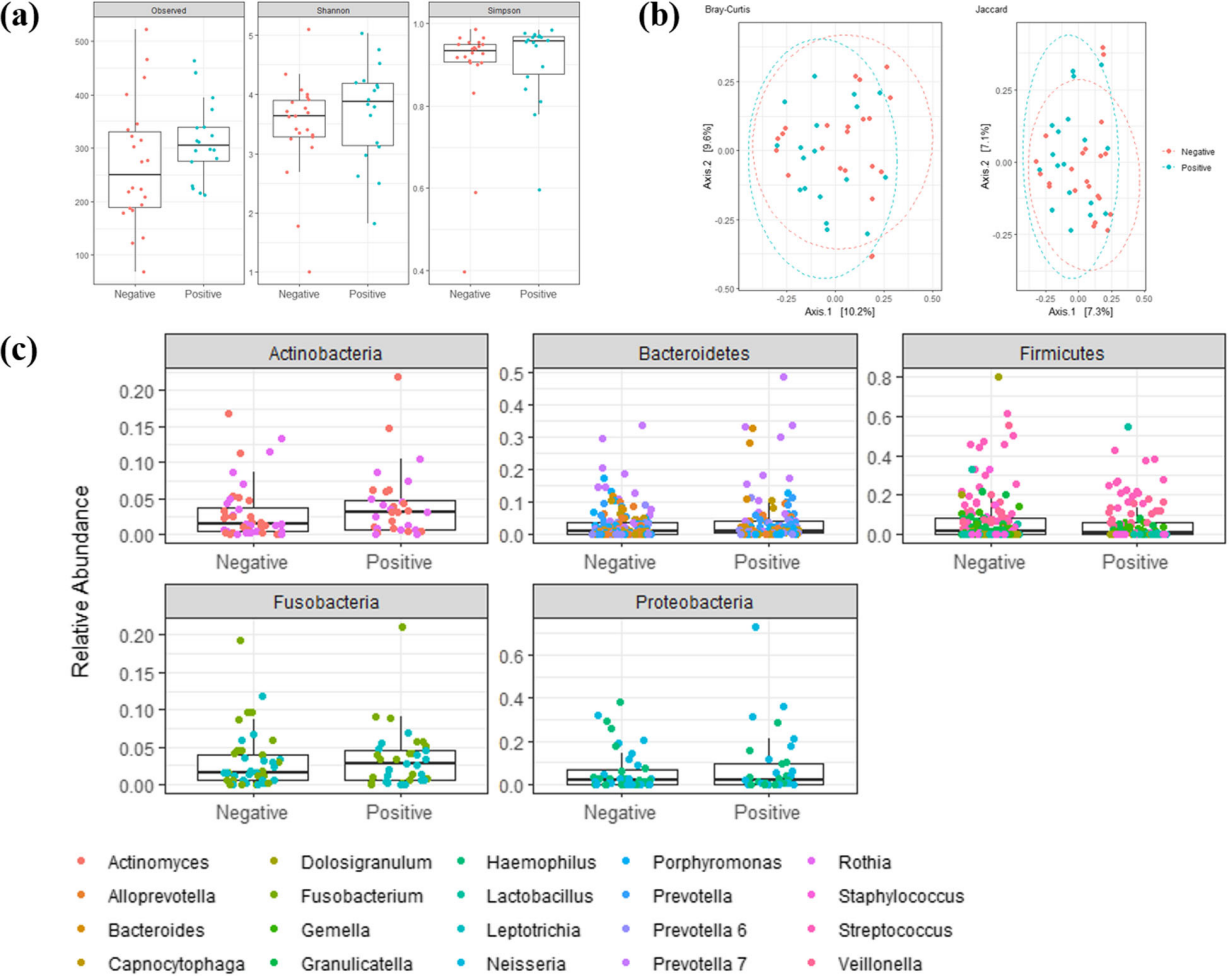
Diversity (a), clustering (b), and taxa abundances (c) of nasopharynx bacterial communities from SARS-CoV-2 positive or negative patients, respectively. In (a) indexes to measure the diversity within samples are shown, in (b) dissimilarity between samples is calculated as the Bray-Curtis or the Jaccard distance, and in (c) taxa represent 20 relatively most abundant genera within the five phyla that compose the nasopharynx bacterial community

The microbiota of the nasopharynx was not different in patients positive for SARS-CoV-2 RNA compared to the microbiota of patients negative for SARS-CoV-2 RNA (Fig. 1). No significant differences between the patient groups in either bacterial richness and diversity (observed species, Shannon index, and inverse Simpson index were assessed; *P* > 0.05 by Wilcoxon signed rank test) or composition (no samples clustering within each group, as visualized by multidimensional scaling; *P* > 0.05 by PERMANOVA) was noticed.

We analyzed the relative abundance of bacterial taxa most represented in both patient groups and did not observe significant differences between the groups. Most sequences in all samples (98% in both SARS-CoV-2 infected and uninfected patients) belonged to five phyla, namely Firmicutes (42 and 51%, respectively), Bacteroidetes (25 and 20%, respectively), Proteobacteria (18 and 16%, respectively), Actinobacteria (8 and 6%, respectively), and Fusobacteria (5 and 5%, respectively). These findings were consistent with the high similarity shared by the bacterial communities in both SARS-CoV-2 infected and uninfected patients.

## Discussion

In this study, SARS-CoV-2 infection was not associated with a different profile of the nasopharynx bacterial community analyzed at COVID-19 diagnosis, which is when the patients had a mild disease. This suggests that no microbiota compositional alterations occurred in response to SARS-CoV-2 or that SARS-CoV-2 was unable to induce these alterations in our patients. Both suggestions lend support to a nasopharyngeal microbiota at least early resilient to SARS-CoV-2 infection.

As nasopharyngeal swabs can be a reasonable proxy for lung samples [20], resilience of the nasopharynx bacterial community would also imply robustness of lung bacterial community in restricting SARS-CoV-2 growth or attachment. This would mirror a lack of effect on the oropharyngeal microbiota in volunteers challenged with H3N2 influenza virus, many of which developed very mild disease [12]. Thus, we did not find bacterial taxa for which relative abundance was significantly higher in SARS-CoV-2 infected patients. Conversely, taxa such as *Dolosigranulum*, *Moraxella*, *Staphylococcus*, and *Streptococcus* (three of which also detected in our study) were found to be enriched in the nasopharynx microbiota of H3N2 influenza virus infected patients compared to healthy control individuals [11].

In one study that analyzed elderly pneumonia patients in comparison with healthy elderly [10], oropharyngeal microbiota profiles differed significantly between the groups, with three of them being significantly associated with pneumonia. Therefore, it is surprising that no published studies until now reported association between upper respiratory tract microbiota and COVID-19, especially in severe disease cases. Here, we studied COVID-19 patients at a time when (mild) symptoms were typical of upper respiratory tract infection and were not affecting the lungs. Consistently, no patients had multifocal ground-glass opacities on chest CT [21], as mentioned above.

The limitations of this study are that SARS-CoV-2 infections were defined by RT-PCR positivity, with no culture results, likely leading to include infections with low infectiousness and the number of analyzed samples was small. Although RT-PCR performed on a nasopharyngeal swab remains the reference diagnostic test [13], patients with negative RT-PCR results could be highly probable COVID-19 cases based on positive chest CT findings [22]. We did not use chest CT to screen for SARS-CoV-2 infection [23] because of awareness that CT findings can be absent, especially in patients with early and/or mild disease [21]. Therefore, we cannot exclude the possibility that false negative RT-PCR results have biased the grouping of patients in our study. Further investigation of SARS-CoV-2 infected patients with detailed viral shedding data and sequential respiratory samples is warranted.

## Data Availability

All data analyzed in this study are available from the corresponding author on request.
